# Economic impact of fetal wastage and common diseases, along with their incidence rates and seasonal variations, at an abattoir in FCT, Nigeria

**DOI:** 10.1371/journal.pone.0310806

**Published:** 2025-02-05

**Authors:** Ibrahim Dauda Dauda, Abdulhakeem Binhambali, Abdurrahman Hassan Jibril, Zainab Oyiza Idris, Farhan Rhidor Akorede

**Affiliations:** 1 Department of Veterinary Public Health and Preventive Medicine, University of Abuja, Abuja, Nigeria; 2 Department of Clinical Science, College of Veterinary Medicine, North Carolina State University, Raleigh, NC, United States of America; 3 Department of Veterinary Public Health and Preventive Medicine, Usmanu Danfodiyo University Sokoto, Sokoto, Nigeria; 4 Department of Theriogenology and production, Faculty of Veterinary Medicine, ABU, Zaria, Kaduna, Nigeria; 5 Department of Veterinary Pharmacology and Toxicology, Usmanu Danfodiyo University Sokoto, Sokoto, Nigeria; University of Uyo, NIGERIA

## Abstract

Infectious diseases and fetal wastage (FW) present major challenges in livestock management, particularly in sub-Saharan Africa, including Nigeria. This study assessed the season variations, incidence rate and economic impact of prevalent diseases and FW at the Kubwa (KB) abattoir in the Federal Capital Territory (FCT) of Abuja, Nigeria. Over a year-long period (January to December 2023), we analyzed 5,779 cattle through daily post-mortem inspections. Disease identification was based on morphological and gross lesions characteristic of the conditions studied. Economic losses from condemned organs and FW were calculated using the formula TEL = N × P × W. The results indicated statistically significant seasonal variations in the incidence of Fasciolosis (FS), Paramphistomosis (PP), and FW, with p-values of 2.52x10^-10, 3.33x10^-9, and 0.003, respectively. In contrast, Tuberculosis (TB), Contagious Bovine Pleuropneumonia (CBPP), Dermatophilosis (DM), Abscess (AB), and Moneziasis (MZ) did not show significant seasonal variation. The total economic impact of condemned organs and FW was 104,348 USD (equivalent to 99,130,600 NGN at the time of the study), representing a considerable threat to food security and substantial economic losses for farmers. Considering the zoonotic potential of some diseases, which can be transmitted to those handling the carcasses, there is a critical need for enhanced inspection protocols, continuous disease surveillance, and timely reporting in slaughterhouses. The notable economic losses from condemned organs also underscore the necessity of implementing pregnancy tests for female animals before slaughter to protect food security and support the nation’s economy. These findings highlight the essential role of abattoirs in improving food security, detecting zoonotic diseases, and bolstering public health and economic stability in low-income countries.

## Introduction

Infectious diseases such as TB, caused by *Mycobacterium bovis*, pose significant threats to both animal and public health, resulting in economic losses from reduced milk production, slaughter condemnations, and trade restrictions [1]. This disease primarily affects the respiratory tract and can be transmitted to humans, especially before milk is pasteurized [2–4]. Bovine tuberculosis (bTB) imposes a major economic burden in Africa and South Asia, with direct economic impacts estimated at $300 million annually and global costs around $3 billion per year [5, 6]. The disease, caused by various members of the *Mycobacterium tuberculosis complex*, spreads through unpasteurized milk, raw meat, and inhalation of droplets from infected livestock. However,the emergence of drug-resistant strains, concurrent infections, and poor living conditions in the developing Nations further exacerbate TB’s impact, leading to substantial global economic losses [7, 8] in these regions. These challenges impede eradication efforts, such as the WHO END TB initiative. The Food and Agricultural Organization (FAO) recommends detecting tuberculosis lesions in slaughterhouses as a form of passive surveillance for bovine tuberculosis [8] and this shows how important abattoir is in disease surveillance and eradication. In Tanzania alone, 1.2% of carcasses are condemned annually due to bovine TB, highlighting significant public health risks [7, 9].

On the other hand, other diseases like CBPP which is caused by *Mycoplasma mycoides subsp*. *Mycoides*, is also a threat to zoonotic transmission and food instability. CBPP is a disease that primarily targets the respiratory system and can be transmitted to humans, leading to severe fibrinous bronchopneumonia, pleural effusion in acute stages, and pulmonary sequestra in chronic cases [10]. CBPP results in high morbidity and mortality rates, causing significant economic losses in the cattle industry due to reduced productivity and increased trade restrictions [11]. Historically, CBPP has been present in Central and Northern Europe since the 16th century and spread globally due to livestock trade, but it has largely been eradicated through stamping-out policies [12], however the situation of eradication is a bit different in some regions in Africa. CBPP remains endemic in sub-Saharan Africa, including Nigeria, where it poses a significant threat to livestock production due to inadequate compensation policies and limited understanding of its epidemiology [13]. In Nigeria, where the cattle population stands at 13.2 million, CBPP, along with other diseases like FS, TB presents a serious challenge to cattle production [14, 15] and threat to farmers whose their livelihood are solely based on animal rearing. The disease’s impact extends to food security, threatening the nation’s primary source of animal protein. Efforts to combat CBPP are hampered by incomplete and irregular vaccination programs, poor funding of veterinary services, and gross under-reporting due to weak surveillance and reporting systems [14].

Also FE is a great concerns to food instability in the developing world including Nigeria, on which several papers has been published on the economic impact on FE. A great example is a study by Nabasirye *et al*. *w*ho noted that FW contributes to approximately 20%-25% of the reduction in livestock populations in sub-Saharan Africa with immediate direct impact on protein diminution [16].

Abattoirs, where these diseases are observed almost daily, play a pivotal role in disease surveillance in livestock, including FW monitoring. They are particularly crucial in detecting common diseases and wastage in cattle through systematic slaughterhouse inspections [17]. According to the FAO Manual for Meat Inspection, effective meat inspection protocols are instrumental in controlling bTB [18] and other significant diseases in endemic regions like Nigeria. This study aims to assess the seasonal variations, incidence rates, and economic impact of prevalent infectious diseases and FW at the KB abattoir in Abuja, Nigeria. It also underscores the necessity of enhancing inspection protocols and disease surveillance to bolster food security and economic stability in the region. Given the ongoing challenges posed by these diseases, there is an urgent need to implement robust surveillance systems and improve abattoir practices to mitigate their impact on public health and the economy.

## Materials and methodologies

**Fig 1:**
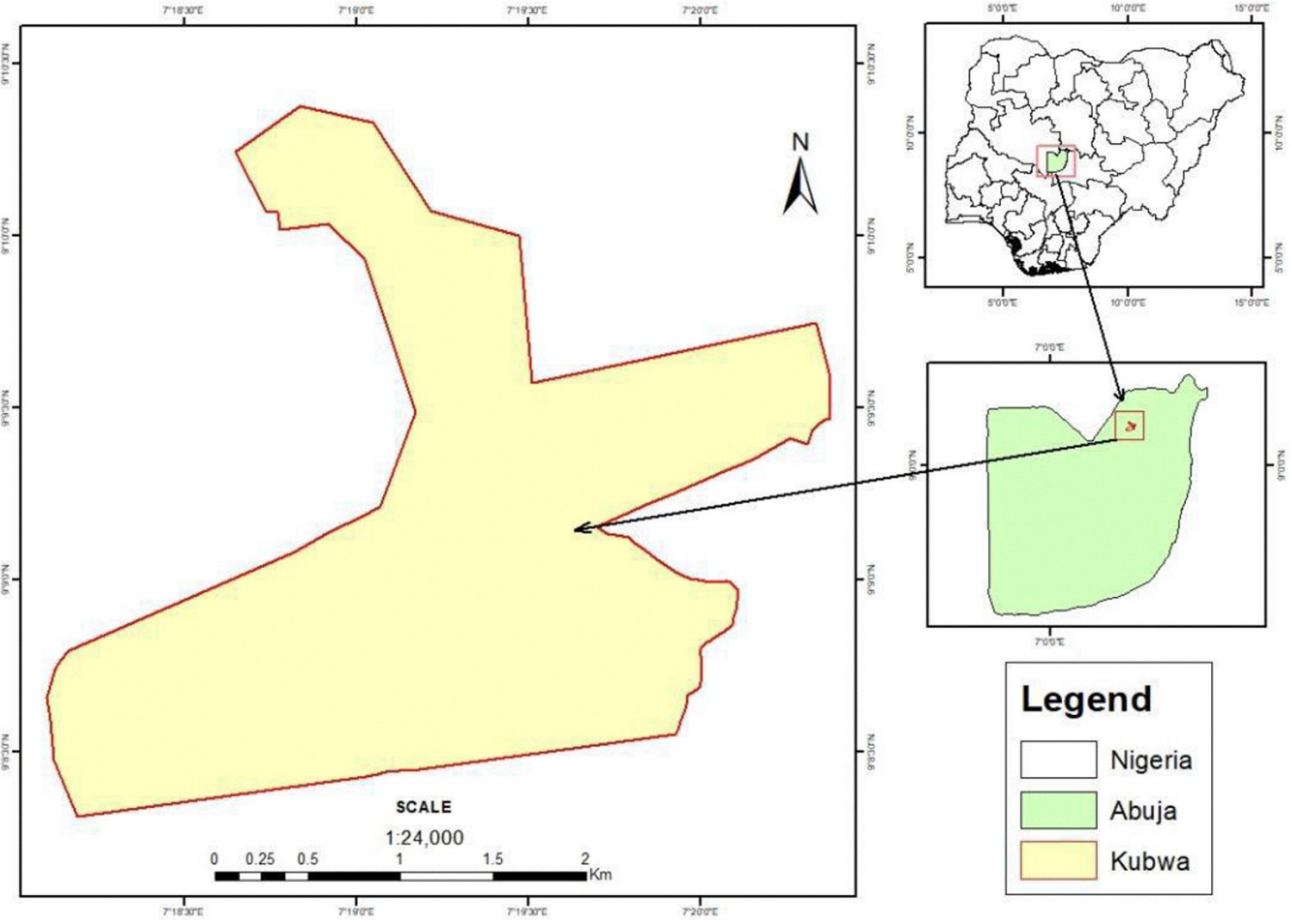
An image showing the study’s geographical information (ArcGIS, Binhambali 2024).

The study was conducted in KB Fig 1, a prominent area council within Nigeria’s capital city, Abuja. Situated in the Bwari Area Council of the Federal Capital Territory (FCT), KB lies in the North-central geopolitical zone of Nigeria, with geographical coordinates at Latitude 9.15190 N and Longitude 7.33330 E. The FCT, which is one of the 37 states in Nigeria, covers a land area of 7,315 km^2^. It consists of six area councils—Abaji, Bwari, Gwagwalada, Kuje, Kwali, and Abuja Municipal Area Council (AMAC)—each experiencing varying climatic conditions. The region is characterized by four distinct seasons: the early dry season (October–December), late dry season (January–March), early rainy season (April–June), and late rainy season (July–September). The mean annual rainfall in the area is approximately 1,389 mm [19], with rainfall spanning around 180 days each year. As of 2016, the population of Abuja was estimated at 3,564,100 [20], with KB’s population around 700,000 as of 2020. The KB abattoir, a significant facility in the region, has an annual average slaughter capacity of 5,700 cattle, with a monthly average of 475 slaughters. This abattoir plays a crucial role in the meat supply chain, impacting both local food security and the regional economy.

### Study design and target populations

A daily analysis of slaughtered cattle was conducted at the KB abattoir from January 2023 to December 2023. Data were collected from daily meat inspection records and post-mortem examinations of the slaughtered trade cattle. The target population included cattle from both nomadic and sedentary pastoral herds within the FCT and neighboring communities. These cattle, aged one year and older, included both sexes (bulls and cows) and represented various breeds such as Bunaji, Bokologi, and Rahaji, which are commonly found in this region. Upon arrival at the abattoir from livestock markets, the animals were typically rested for at least 24 hours before slaughter. Post-mortem investigations were then conducted, focusing on the detection of diseases through gross observations of various organs.

### Sample size determination and sampling method

The sample size was determined using the Open Source Epidemiologic Statistics for Public Health (OpenEpi 2.3) software, with parameters set at 50% power, a 5% margin of error, and a 95% confidence level [21]. Based on the recorded data, some results were presented using descriptive statistics, and where applicable, the results were statistically analyzed using a one-way analysis of variance (ANOVA) parametric test. The recorded epidemiological factors, such as age and sex, were compared using the chi-square (χ^2^) test [22].

### Records analysis and examination process

Records of slaughtered animals, diseases, and conditions for organ condemnations identified through gross pathology were analyzed for one year (2023). Disease identification was based on morphological and gross lesions characteristic of the conditions studied as validated by Awah-Ndukum *et al*. (2007) [23] and Cadmus (2007) [24] in their various studies. The validation of the diseases in this study was done by the veterinarians and trained meat inspectors attached to the abattoir on a daily basis. However, due to lack of the laboratory facilities at the abattoir site, a major concerns for most of the abattoir in the country, we were not able to do microbial and parasitic isolation, as well as the molecular characterization of the organisms and this was the major limitation of this study. Qualitative data were manually analysed, and percentages for various causes of condemnations were calculated.

The study was cross-sectional, conducted daily from 6:00 am to 9:30 am, Monday to Sunday. Ante-mortem (AM) is done to assess the animals’ condition and treatment to ensure compliance with animal welfare regulations while detecting any signs of disease or injury that could compromise the safety and quality of the meat after slaughter, as well as post-mortem (PM) examinations to examine the carcasses and organs of slaughtered animals for signs of disease, contamination, or other abnormalities to ensure that the meat is safe and fit for human consumption.

Despite inadequate restraint facilities, AM inspections were done remotely and hands-on in the lairage. PM examinations focused on the skin, lungs, liver, rumen, kidney and other internal organs to identify gross lesions indicative of the studied diseases. Organs were separated from the carcasses and examined on the inspection slab in the slaughter hall by seven veterinary trainees and three animal scientists, supported by five designated abattoir meat inspectors who are Veterinarians that are licensed to do carcass inspection at the abattoir. The study recorded the date of inspection, the sex of the cattle, and the presence or absence of lesions on the organs, examining a total of 5779 animals.

### Detailed post-mortem examination

**Fig 2:**
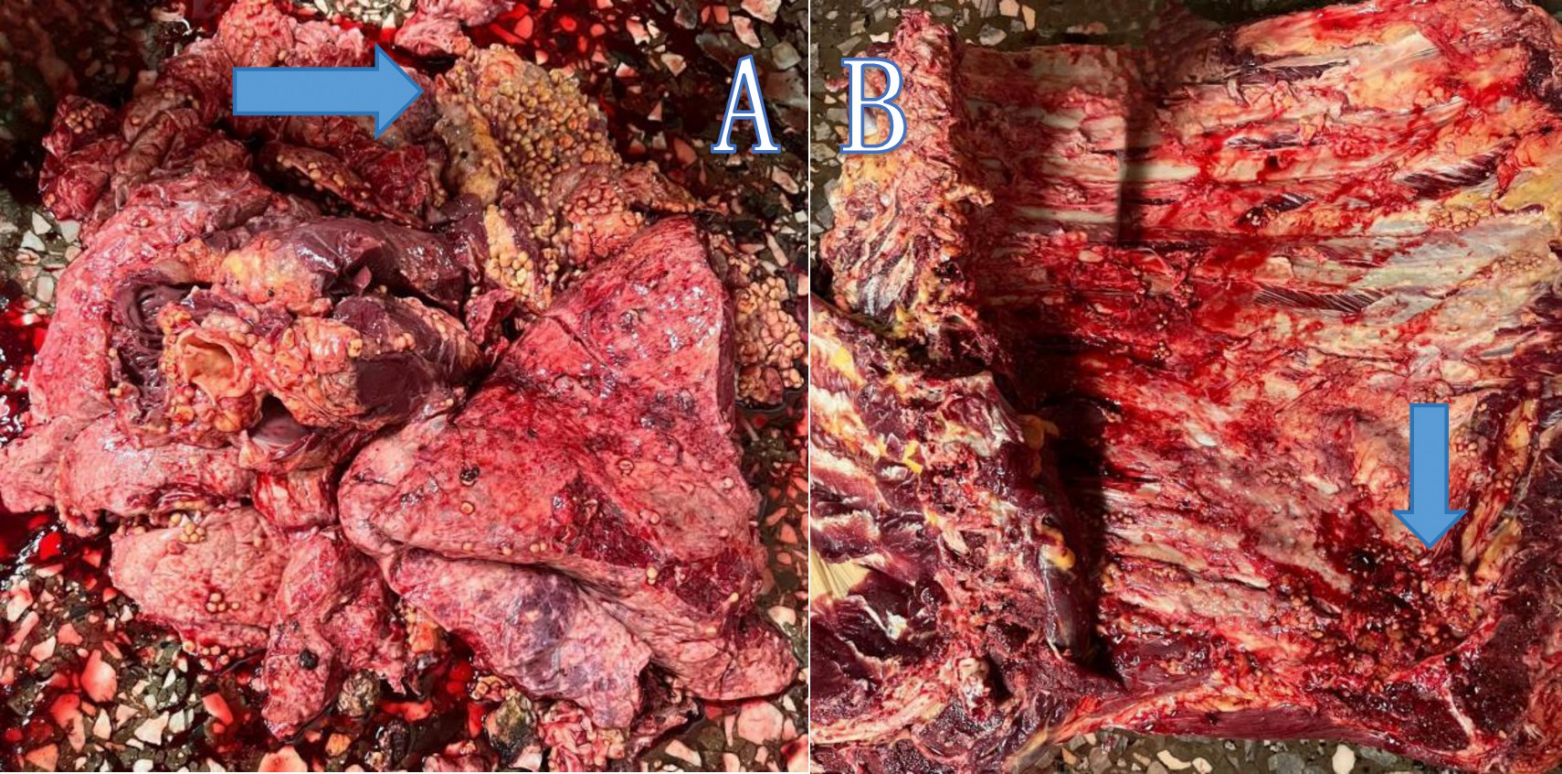
Organ and tissue affected by tuberculosis. An arrow showing a lung of an animal infected with tuberculosis, with distinct miliary tubercles. (B) Section of the thoracic wall from the same infected animal, with tubercles visible within the thoracic wall.

**Fig 3:**
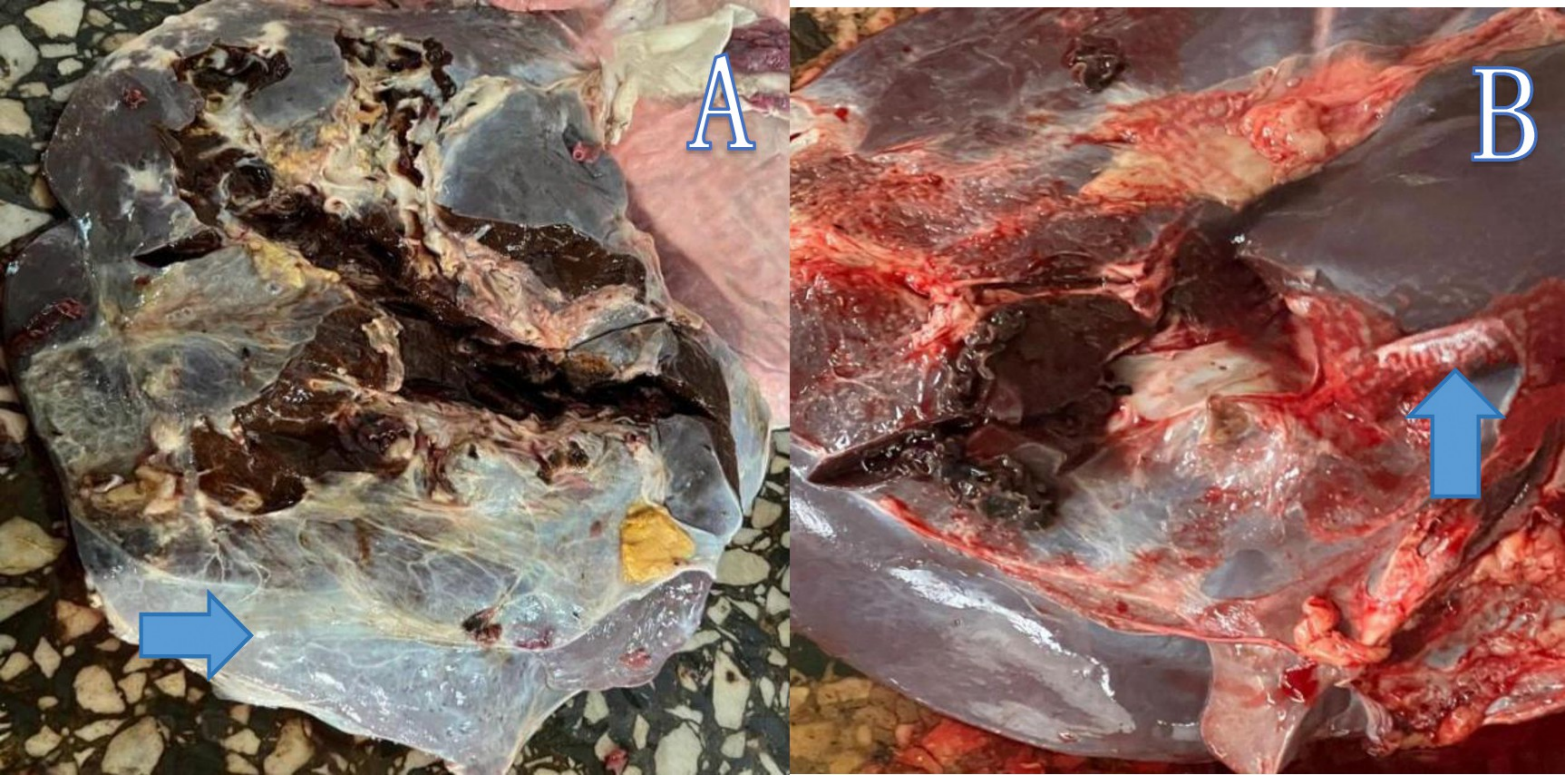
Condemned liver. (A) An arrow showing a liver of an animal infected with fasciolosis, displaying generalized fibrotic tissue. (B) An arrow showing liver of an infected animal, showing the presence of *Fasciola hepatica* within the liver bile duct.

Post-mortem examinations, particularly of the lungs Fig 2A, thoracic wall Fig 2B and liver Fig 3A and 3B, were carried out in the well-lit ’red offal room’ at the liver inspection station. The plucks were carefully removed, and the organs were meticulously examined for signs of tubercles, fibrosis, color changes, fatty changes, and fasciola infestation Fig 3B. Although the severity of fasciolosis was graded—ranging from mild (one bile duct affected), moderate (2–4 bile ducts affected), to severe (more than four bile ducts affected)—this grading was not utilized in the study. Each liver was trimmed at the portal zones to expose the bile ducts and assess fasciola infestation. Infested liver sections were trimmed and discarded, while healthy portions were approved for consumption. Livers that were severely affected were entirely condemned and weighed in kilograms. Tuberculosis-affected lungs were differentiated from those affected by CBPP based on the lesions observed. Descriptive statistics were computed for all examined diseases. This meticulous approach ensured accurate detection and grading of diseases, providing valuable data for the study.

### Data analysis and economic loss calculation

**Fig 4:**
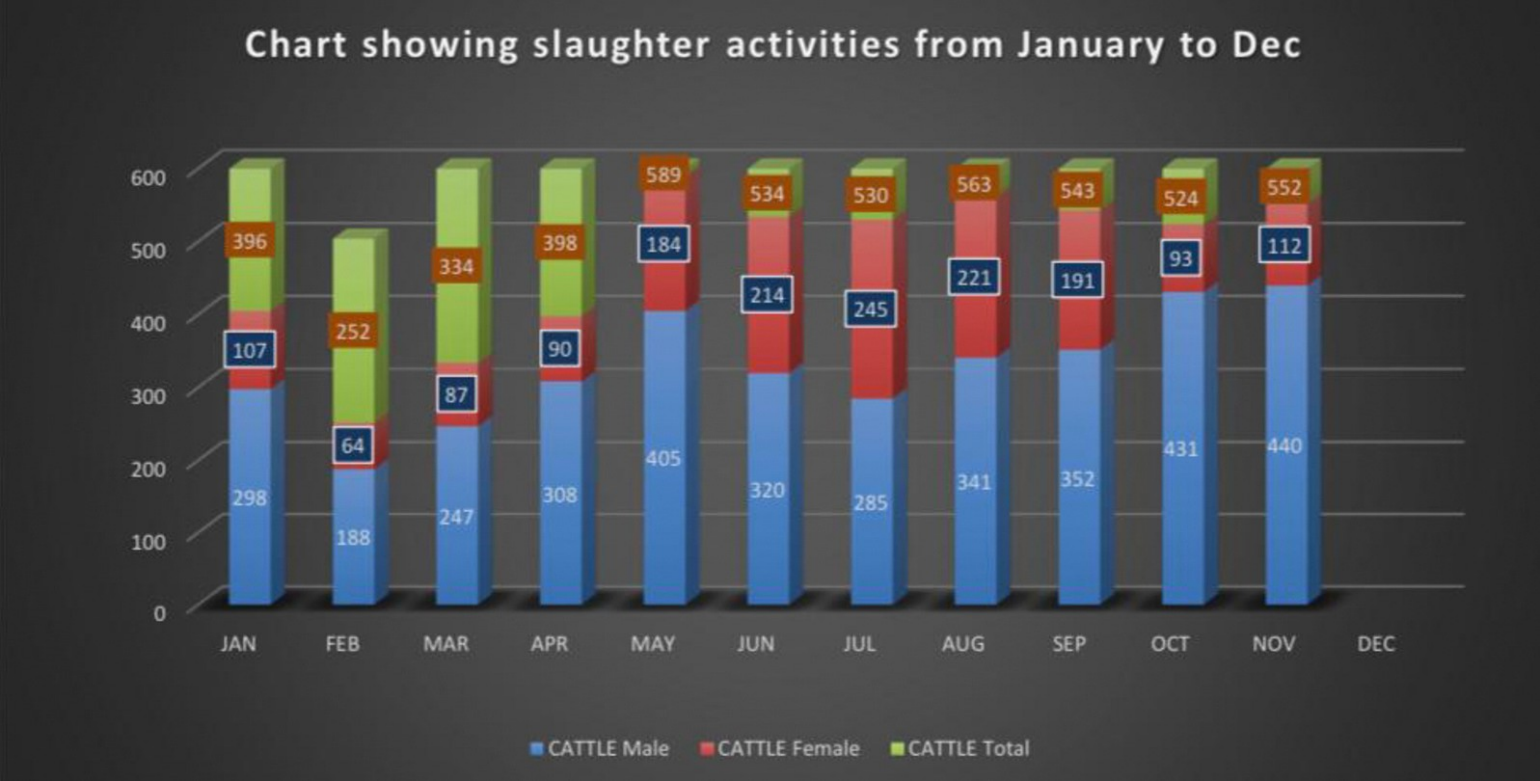
A graph showing the slaughter activities of Kubwa abattoir from Jan-Dec.2023, which includes the sex distribution of the slaughter cases.

The collected data were summarized using Microsoft Excel 7 (Microsoft Office, Redmond, USA) and analyzed with OpenEpi version 2.3.1 software [21]. Descriptive statistics were used to represent the proportions of the data obtained. The study focused on the prevalence of common diseases such as TB, CBPP, FS, PS, MZ, and FW. The variables analyzed included intrinsic cattle characteristics (such as sex) Fig 4 and extrinsic factors (such as season period and geographical location). Significant factors were identified through univariate analysis using Chi-square tests [25], and these were further examined using multivariate analysis through stepwise backward logistic regression to control for confounding variables and test for effect modification.

The total economic loss was calculated by summing the value of condemned livers and lungs separately, using the NGN to USD exchange rate for 2023. The formula applied was: TEL = N × P × W, where TEL represents the total economic loss, N is the number of condemned livers/lungs, P is the average price of the liver/lung in USD per kilogram, and W is the average weight of the liver/lungs in kilograms. This formula was validated by Alhaji *et al*. [26] in their study. The average weight of cattle livers was determined to be 3.2 kg, while lungs averaged 3.0 kg, based on regular inspections. The average price per kilogram for liver was 5.0 USD (NGN 4,750/kg), equating to 16.0 USD (NGN 15,200) per liver. For lungs, the average price was 3.5 USD/kg (NGN 3,325/kg), or 10.5 USD (NGN 9,975) per lung, according to interviews with local butchers and meat sellers. The exchange rate of NGN 950 to 1 USD, as reported by the Central Bank of Nigeria in 2023, was used for these calculations (CBN, 2023).

### Ethical statement

The study protocol received approval from the FCT Department of Public Health and Preventive Medicine Development Research Ethics Committee with the approval number of FCTA/PBHS/RA005. Prior to sample collection, advocacy visits were conducted by the abattoir’s Disease Reporting Officer to engage with the abattoir management.

## Results

**Fig 5:**
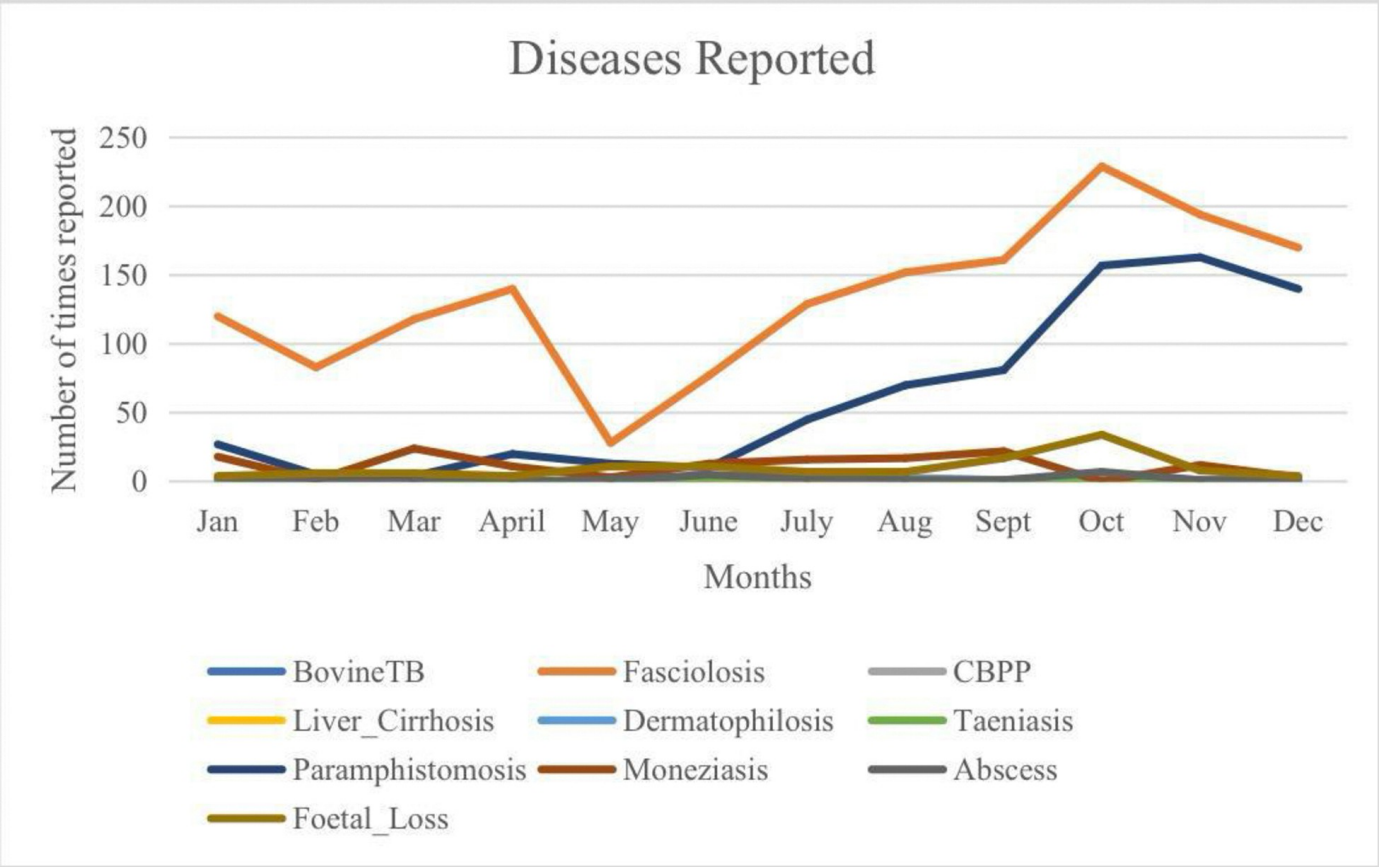
A chart illustrating the incidence rates and occurrences of each reported disease at Kubwa Abattoir from January to December 2023.

The seasonal incidence rates of various diseases in cattle were studied Fig 5, with the results presented in Table 1. For bTB, 10 cases were positive during the wet season and 4 during the dry season, with 3,670 and 2,095 negative cases, respectively (p = 0.54). CBPP showed 13 positive cases in the wet season and 6 in the dry season, with 3,667 and 2,093 negative cases, respectively (p = 0.66). FS had 916 positive cases in the wet season and 685 in the dry season, with 2,764 and 1,414 negative cases, respectively (p = 2.52×10⁻^1^⁰). Liver cirrhosis recorded two positive cases in both seasons, with 3,678 and 2,097 negative cases, respectively (p = 0.57). DM showed 16 positive cases in the wet season and 4 in the dry season, with 3,664 and 2,095 negative cases, respectively (p = 0.13). Taeniasis had two positive cases in the wet season and 3 in the dry season, with 3,678 and 2,096 negative cases, respectively (p = 0.27). PP showed 396 positive cases in the wet season and 339 in the dry season, with 3,284 and 1,760 negative cases, respectively (p = 3.33×10⁻⁹). MZ recorded 82 positive cases in the wet season and 58 in the dry season, with 3,598 and 2,041 negative cases, respectively (p = 0.20). Abscesses were identified in 17 positive cases during the wet season and 5 during the dry season, with 3,663 and 2,094 negative cases, respectively (p = 0.18). FW was observed in 91 positive cases in the wet season and 28 in the dry season, with 3,589 and 2,071 negative cases, respectively (p = 0.003).

**Table 1:** Bi-seasonal occurrence of various diseases in slaughtered at the Kubwa abattoir from January to December 2023.

Diseases	Positive (Wet_season)	Negative (Wet_season)	Positive (Dry_season)	Negative (Dry_season)	*p*-value
Bovine TB	10	3670	4	2095	0.54
CBPP	13	3667	6	2093	0.66
Fasciolosis	**916**	2764	**685**	1414	**2.52x10** ^**-10**^
Liver cirrhosis	2	3678	2	2097	0.57
Dermatophilosis	16	3664	4	2095	0.13
Taeniasis	2	3678	3	2096	0.27
Paramphistomosis	**396**	3284	**339**	1760	**3.33x10** ^**-9**^
Moneziasis	82	3598	58	2041	0.20
Abscess	17	3663	5	2094	0.18
Foetal Loss	**91**	3589	**28**	2071	**0.003**

The table above indicates no statistically significant relationship between the occurrence of the outlined diseases and seasonal variations, except for FS, PS, and FW. The p-values for these diseases are 0.00000000025, 0.0000000033, and 0.003, respectively. Their p-values are less than 0.05, indicating a statistically significant association between seasonal changes and the occurrence of these diseases and FW. This suggests a statistically significant association between seasonal changes and the incidence of these diseases. This means that, for all the other diseases, we accept our null hypothesis, while for FS, PS and FW, we rejected the null hypothesis and accept the alternative hypothesis This alternative hypothesis posits that there is a significant association between the occurrence of these diseases and seasonal variations during the study year.

## Discussion

The abattoir plays a critical role in gathering data on livestock pathogens and food wastage, which is vital for effective risk assessment [8, 9] and economic sabotage. Surveillance at slaughterhouses is essential for detecting bTB cases, as highlighted by Pascual-Linaza *et al*. [17] and wastage such as meat through condemnation and FW. Adherence to proper meat inspection protocols and effective surveillance are crucial for controlling this situation especially in endemic areas such as Nigeria [9]. In this study, we identified 14 cases of bTB, with 10 cases occurring during the rainy season and 4 during the dry season. Although our results did not show a statistically significant difference between the wet and dry periods for this particular disease, however, the higher incidence during the rainy period may be attributed to the increased number of animals slaughtered during this time, as documented earlier in this study. The incidence rate observed in our study is consistent with findings from Cadmus *et al*. [24], who reported a bTB incidence rate of 7.95%. Similarly, a report from Turkey by Yibar *et al*. reported 1.32% [27] which is closely similar to a study from Algeria by Dergal et al. [22] that reported an incidence rate of 2.73% and like with a study from Morocco by Hamid *et al*. [28] who reported 4.6% incidence rate, these results aligns with the lower rate observed in our research. The lower incidence rate in our study could also be attributed to increased awareness among farmers regarding the zoonotic transmission of bTB, which may lead them to avoid sending visibly diseased animals to the abattoir. Additionally, the fear of having their animals condemned by abattoir veterinarians may further deter farmers from presenting infected animals.

The economic impact of condemned lungs due to bTB at our study site was approximately 147 USD (NGN 139,650). This estimate is based on a price of 3.5 USD per kilogram and an average lung weight of 3.0 kg per animal, translating to 10.5 USD (NGN 9,975) per animal.

The results for bTB and CBPP revealed low incidence rates and similar seasonal trends. In this study, we recorded 19 cases of CBPP, with 13 cases occurring during the wet season and 6 during the dry season. No animals presented with mixed infections of bTB and CBPP or other diseases. According to Alhaji *et al*. (2016), the occurrence and distribution of CBPP in cattle herds are influenced by various intrinsic and extrinsic factors, including intermittent infections, age, genetic constitution, crowding, climatic conditions, and stress from transportation and handling [29]. These factors significantly affect the outcome of CBPP infections in herds. The most recent report of CBPP incidence in Nigeria recorded 489 cases, with 96 deaths, 221 slaughters, and 9 animals destroyed [15]. However, this figure may not fully reflect the actual infection rate due to underreported cases, which can result from some farmers’ nonchalant attitudes toward disease reporting. Nonetheless, Similar study was done in Ethiopia by Edo *et al*. [30], where they recorded 25.7% and 24.8% condemnation rate for both liver and lungs. Although, our study identified 19 cases of CBPP, which were relatively few and may reflect the regional scope of our data. Additionally, farmers may avoid selling sick animals due to potential loss of profit, similar to the reluctance seen with bTB cases. Inadequate compensation mechanisms during disease outbreaks may also contribute to this trend.

In 1996, the economic cost of CBPP in Nigeria’s northern livestock industry was estimated at US$1.5 million [31]. However, in this study, the total estimated economic cost of CBPP in pastoral herds was approximately to a sum of USD 2,802.50 (NGN 2,662,375), or USD 147.50 (NGN 140,000) per head of cattle based on total condemnation policy. This figure is significantly lower than the economic loss reported by Alhaji *et al*. [26], who estimated the loss due to CBPP in Niger State, one of the north-central states in Nigeria, to be around USD 294,800.30. Differences in data considerations likely contribute to the discrepancies observed in our valuation. Additionally, we identified 22 abscesses, with 17 occurring during the wet season and 5 during the dry season. These abscesses are frequently associated with bTB or CBPP cases, reflecting the seasonal trends of these diseases. This observation may be linked to the pathological processes associated with both bTB and CBPP.

Our study identified 916 positive cases of FS during the wet season and 685 cases during the dry season. Of these, 671 cases involved partial liver condemnation, and the total number of condemned livers reached 930, yielding an incidence rate of 27.7%. This finding is consistent with Odeniran *et al*. (2020) [32], who reported a fasciolosis prevalence of 18.3%. Our study’s total liver condemnation rate was 58.1%, which is significantly higher than the 11.1% reported in their study, based on an annual slaughter rate of 10.5% from a total cattle population of 20 million. Similarly, Isah *et al*. (2019) [33] observed infection rates of 48.5% and 46.9% at two of their seven study sites, with a significant *p-*value of 0.05, aligning closely with our results. Our findings also align with research from north-eastern Nigeria by Shinggu *et al*. [34] and reports from Mexico by Hernández-Hernández *et al*. [35], where the general incidence rates of liver flukes were approximately 25% and 28.6% for male and female cattle, respectively [34]. Additionally, Hernández-Hernández *et al*. reported incidence rates of 32.3% in 2018 and 41.7% in 2019 [35]. These high incidence rates corroborate the findings of Cadmus *et al*. [24], who reported a fasciolosis incidence rate of 20.28%. However, the incidence rate in our study is slightly higher than that reported by Yibar *et al*. [27] in Turkey, which found a rate of 2.028%, despite a comparable number of cattle being assessed. It is worth noting that their study was conducted over six months, whereas our study spanned an entire year.

The varying incidence rates of fasciolosis can be attributed to multiple factors. Effective control strategies generally lead to lower prevalence, while favorable local environmental conditions, particularly during the rainy season, promote the development of the snail intermediate host, thereby enhancing the parasite’s reproductive cycle [24]. In our study, seasonal changes significantly impacted the occurrence of bovine FS among cattle slaughtered at the KB abattoir. The highest infection rates were observed during the early rainy period, with a subsequent decline in the late rainy period and a further decrease into the early dry period, as shown in Fig 5. This finding aligns with Njoku *et al*. (2011) [36], who reported a high incidence of bovine FS in cattle slaughtered during the rainy season in Imo State, Nigeria. The increased burden of infections during the rainy season is likely due to the proliferation of snails in grazing areas. Cattle grazing in fields, near riverbanks, and around water bodies are more susceptible to contracting the infective stage of FS, leading to a peak in infections by the late rainy season following an initial rise in the early rainy season. Additionally, grazing near seasonal extensions of rivers and lakes significantly heightens the risk of fasciola infections [37].

Our study revealed a high burden of FS during the dry season, with an incidence rate of 685 cases. This result contrasts with findings by Yatswako *et al*. (2017) [38] and Kuchai *et al*. (2011) [39], who reported lower incidence rates during the dry season. We also observed two cases of liver cirrhosis in both the dry and wet periods, which occurred alongside some FS cases. The pathology of liver cirrhosis may be linked to the damage caused by the migration of liver flukes. Additionally, our study identified five cases of taeniasis in both wet and dry periods. This finding diverges from reports by Okafor *et al*. [40] and *Okolo et al*. [41], who documented higher incidence rates of taeniasis at 26.14% and 26.2%, respectively, suggesting a lower rate in our study.

From an economic standpoint, we estimated a substantial loss of 18,235 USD (NGN 17,323,250) due to condemned livers at our study site. This estimate was based on a price of 5.00 USD per kilogram of healthy fresh liver, with an average liver weight of 3.2 kg per condemned liver (930 total), valuing each at16 USD (NGN 15,200). For partial condemnations (671 total), which averaged 1 kg each, the value was 5.00 USD per partial condemnation (NGN 4,750). Our economic findings are consistent with the impact reported by Yatswako *et al*. (2017) [38], with total number of 621 cattle tested positive [38] and Odeniran *et al*. study [32] with annual loss of 26.02 million USD from cattle population of 20 million as well as Elelu *et al*. (2018) [42] study who reported a staggering total economic loss of cattle to FS in Nigeria, estimated at £32.5 million and documented a total loss of 776,832 USD from 47,931 condemned livers in their study from 2005 to 2015. This economic loss is also similar to a recent study from Europe by Ciui *et al*. [43] which revealed the economic loss to be EUR 360,316.9 for organs condemnation alone in their study, with greatest loss from liver condemnations with financial loss of EUR 302,737.5. Although our estimated economic loss was lower than the mentioned figures, possibly due to differences in dataset size and cattle head counts, our findings still indicate a concerning upward trend in the economic impact of FS, aligning with the trends observed in previous studies.

DM exhibited an exceptionally low incidence rate in our study, with only 20 positive cases out of 5,779 examined. Of these, 16 occurred during the wet season and 4 during the dry season. The low incidence rate of DM may be due to farmers’ hesitancy to sell animals with visible skin conditions like DM. Farmers might prefer to treat these animals before sale to avoid reduced profits, similar to practices observed with other diseases. Additionally, seasonal variations could play a role, as infectious agents often thrive better in the wet season due to higher humidity, which facilitates rapid multiplication compared to the dry season. However, for PP, we recorded 735 cases, resulting in an incidence rate of 12.7%, with 396 cases during the wet season and 339 during the dry season. The relatively consistent incidence rates across both seasons might be attributed to several factors. Adult paramphistomes generally do not cause overt disease, and large numbers can be present without immediate symptoms, while immature paramphistomes can remain attached to the duodenal and ileal mucosa for extended periods [44]. Additionally, inadequate control measures by farmers in our study area may contribute to this consistency, as some farmers may only take action once their herds are visibly affected. The incidence rate observed in this study differs from a study in Pakistan, which reported a much higher incidence rate of 56.25% in ruminants, with cattle showing the highest rates [45]. The Pakistani study emphasized that the incidence of PP spp. is influenced by specific climatic and meteorological factors, with climate change potentially altering parasite infection patterns and affecting hosts [46]. Extrinsic factors such as temperature, humidity, rainfall, water velocity, and habitat stability also impact the production costs associated with paramphistomum infections [45]. These factors affect the metabolic systems of both the parasite and its host, disrupting the growth, survival, and reproduction of snails, which are intermediate hosts for the parasite [47]. Also, studies in France [48], Spain [49], Ireland [50], and England [51] reported prevalence rates of 20%, 6.2%, 52%, and 25%, respectively. Aside the Spain that also recorded lower incidence rate like our study, the elevated rates of other European countries could be attributed to distinct climatic conditions in these regions compared to ours, explaining the differences in findings. Notably, no research has documented economic losses from Paramphistomosis infections, likely because rumen damage is typically minor and does not lead to total organ condemnation except on few scenarios.

Our study recorded approximately 140 cases of moneziasis across both wet and dry periods, resulting in an incidence rate of 2.4%. This finding is consistent with a study conducted in Serbia by Pavlovic *et al*. (2022) [52], which reported a similar incidence rate of 3 to 5%.

A PM examination of 1,608 female cattle at the KB abattoir revealed a significant incidence of pregnancy and FW, with a rate of 2.06%. Most of the slaughtered pregnant females exhibited normal breeding potential, with few genital disorders and frequent PM pregnancies. However, this contrasts with a study from Ethiopia, which reported a much higher FW rate of 39.8% [53]. Similarly, a study on goats by Ugwu *et al*. (2023) [54] in Enugu, Nigeria, found that 35.5% of slaughtered female goats were pregnant, resulting in the loss of 907 foetuses over six months. This result was different from what Njoga *et al*. *p*ublished from a study in Southeastern Nigeria with an incidence rate of 58.8% of pregnant cow slaughtered during the dry season [55].

The total economic loss due to FW in our study was estimated at 83,300 USD (NGN 79,135,000), calculated at 700 USD per head, representing the potential earnings if the foetuses had been born, raised to maturity, and slaughtered. This figure closely aligns with the economic loss reported by Ugwu *et al*. [54], which was approximately 83,390 USD. Our findings is closely similar to the study by Nabasirye *et al*. who reported FW to about 13,224 USD monetary losses at birth, 31,849 USD loss at weaning and 57,0896 loss at maturity [16] for three species of animals namely (Bovine, porcine and Ovine).The indiscriminate slaughter of pregnant animals contributes to this loss, driven by factors such as increasing domestic meat demand, economic constraints faced by farmers, inadequate slaughter regulations, and various biological, social, and climatic influences [56, 57].

A comparison with European data published in 2017 by the European Food Safety Authority Panel on Animal Health and Welfare, as reported by More *et al*., highlights stark differences, with median percentages of all mature female animals slaughtered while pregnant estimated at 16%, 11%, 6%, 10%, and 4% for dairy cows, beef cattle, pigs, sheep, and goats, respectively [58]. Given the substantial number of pregnant animals slaughtered and the consequent FW observed in this study, there is an urgent need to amend and rigorously enforce the Meat Edict of 1988, as noted by Ugwu *et al*. [54]. Proposed amendments might include making pregnancy diagnosis a mandatory component of the post-mortem inspection protocol for all female animals intended for slaughter in the study area, especially considering that many farmers at our study site avoid AM screening due to the risk of outright rejection of their animals.

## Conclusion and limitation

This study highlights the significant challenges posed by ID and fetal FW in livestock management within sub-Saharan Africa, particularly Nigeria. The findings from a year-long analysis at the KB abattoir reveal substantial seasonal variations in disease incidence and underscore the severe economic losses resulting from condemned organs and FW. This study revealed the critical need for improved inspection protocols, continuous disease surveillance, and the implementation of pregnancy tests for female animals before slaughter. Addressing these issues is essential for enhancing food security, detecting zoonotic diseases, and supporting public health and economic stability in low-income countries. The study’s primary limitation was the inability to perform microbial and parasitic isolations or molecular characterization of diseases due to inadequate laboratory facilities at the abattoir. Additionally, while gross lesion observations were used for disease identification, the lack of advanced diagnostic tools could affect some of the findings of this study.
